# Supplementary material for: Wide-swath altimetry maps bank shapes and storage changes in global rivers
Source: Nature. 2026 Mar 4;651(8106):666–71. doi: 10.1038/s41586-026-10218-y (PMC12999504; doi:10.1038/s41586-026-10218-y)
Supplement: Supplementary file 1 — This supplement presents additional information on the MeanDRS river storage model simulation in section 1, and on scaling the observed RSAs from the existing filtered SWOT dataset to the more comprehensive SWORD database in section 2. It also outlines the current observational and methodological limitations associated with SWOT data and our methodology in section 3. In addition, this supplement describes a sensitivity analysis of the SWOT width and elevation uncertainties used as inputs in the hypsometric method in section 4. Finally, it provides an outlier analysis of the resulting SWOT-derived ΔRSA magnitude estimates in section 5, offering insights into the reliability of river storage variability estimates with regard to the characteristics of the observation network. [file 41586_2026_10218_MOESM1_ESM.pdf]

nature portfolio
https://doi.org/10.1038/s41586-026-10218-y

Supplementary information

Wide-swath altimetry maps bank shapes and storage changes in global rivers

In the format provided by the authors and unedited

Supplementary Information For

Wide-Swath Altimetry Maps Bank Shapes and Storage Changes in Global Rivers

Cerbelaud A.[1,]*, Wade J.[1,]*, David C.H.[1], Durand M.[2], Frasson R.P.M.[1], Pavelsky T.[3], and Oubanas, H.[4]

[1] Jet Propulsion Laboratory, California Institute of Technology, Pasadena, CA, USA
[2] School of Earth Sciences, The Ohio State University, Columbus, OH, USA
[3] Department of Earth, Marine and Environmental Sciences, University of North Carolina, Chapel Hill, NC, USA
[4] Institut National de Recherche pour l'Agriculture, l'Alimentation et l'Environnement, UMR G-eau, Montpellier, France

* These authors contributed equally to this work.

Correspondence: A. Cerbelaud, arnaud.cerbelaud@jpl.nasa.gov; C.H. David, cedric.david@jpl.nasa.gov

This supplement presents additional information on the MeanDRS river storage model simulation in section 1, and on scaling the observed river storage anomalies (RSA) from the existing filtered Surface Water and Ocean Topography (SWOT) dataset to the more comprehensive SWORD database in section 2. It also outlines the current observational and methodological limitations associated with SWOT data and our methodology in section 3. Additionally, this supplement describes a sensitivity analysis of the SWOT width and elevation uncertainties used as inputs in the hypsometric method in section 4. Finally, it provides an outlier analysis of the resulting SWOT-derived ΔRSA magnitude estimates in section 5, offering insights into the reliability of river storage variability estimates with regards to the characteristics of the observation network.

1. MeanDRS river storage simulations

The Mean Discharge Runoff and Storage (MeanDRS, Collins et al.[1]) dataset is a 30-year global discharge and river storage open-access simulation bias-corrected by in situ gauges using long-term inverse routing. The simulations provided by MeanDRS represent the most recent, publicly available, global estimates of river storage variability and the most readily comparable dataset to our SWOT RSA estimates. MeanDRS uses a simplified version of the Routing Application for Parallel computation of Discharge[2]

(RAPID) to route land surface model runoff through ~3 million river reaches provided by the Multi-Error-Removed Improved Terrain (MERIT) Hydro (v0.7) Basins (v0.1) dataset (MERIT-Basins[3–6]). MeanDRS discharge simulations are transformed into reach-specific estimates of river storage by linearly scaling discharge by the Muskingum time parameter *k* under steady-state assumptions[1]. As the time parameter *k* and the related flow wave celerity of rivers is poorly constrained globally, Collins et al.[1] estimate river storage under three scenarios, with time parameter *k* values corresponding to mean residence times of 1.87 h (low volume), 3.27 h (medium volume), and 4.67 h (high volume) in ~9.4 km reaches, or equivalently flow wave celerity values of 1.39 m/s (low volume), 0.79 m/s (medium volume), and 0.56 m/s (high volume). The resulting MeanDRS river storage time series are scalar multiples of each other dependent on the choice of *k* and are expected to bound true global river storage based on existing knowledge of river celerity.

2. Scaling SWOT-observed storage anomalies

As we compute SWOT-derived RSA for only 73% of type 1 and type 5 SWORD reaches, limited to locations with sufficient valid observations, the resulting global anomalies are not fully representative of the true total global river storage variability. With the accumulation of additional SWOT observations over time and ongoing enhancements to data processing algorithms, a greater proportion of observations are expected to meet quality standards in the future. To account for the anticipated advancement of the SWOT data products, we present an approach to scale the storage estimates obtained from the existing SWOT dataset to estimate the potential RSA that could be observed if comprehensive, high-quality data were available for all SWORD reaches.

In a similar manner to our initial comparison of SWOT RSA to MeanDRS simulations, we retrieve MeanDRS storage time series at MERIT-Basins reaches corresponding to all Type 1 and 5 SWORD reaches, rather than just the SWORD reaches where we compute RSA. This “full SWORD” MeanDRS time series provides a simulated reference for the RSA that SWOT could observe within each basin under the assumption that all SWORD reaches are reliably measured and unaffected by quality filtering constraints.

The relative difference between the "full SWORD" MeanDRS RSA and the anomaly corresponding to SWORD reaches where we compute storage represents the portion of a basin's storage anomaly that remains unobserved as a result of data quality filtering. We scale the observed SWOT RSA in each basin by obtaining the least-squares scaling factor a that best fits the function $Y = aX$, where Y is the MeanDRS RSA corresponding to all Type 1 and 5 SWORD reaches and X is the MeanDRS RSA corresponding to SWOT-observed SWORD reaches. The scaling factor a is determined by:

$$a = \frac{\sum XY}{\sum X^2} \tag{1}$$

We compute the SWOT-derived RSA scaled to all SWORD reaches in each basin by multiplying the observed anomaly by a. This process results in a global increase in the ΔRSA magnitude of 74 km^3 (+23.6%), from 313 km^3 to 387 km^3, with regional variability in the magnitude of scaling (Fig. S1-S3). As we are unable to compute storage at roughly 27% of SWORD reaches globally (Type 1 and 5), the magnitude of scaling produced by our approach matches our expectations.

Even after scaling the SWOT-derived RSA to the expected anomaly at all SWORD reaches, the resulting storage anomaly is still not representative of total global river storage variability. While SWORD is designed to map global rivers wider than 30 m[7] and SWOT is expected to produce reliable WSE and width measurements for rivers wider than 50-100 m, the mission is not capable of observing all global rivers. Using storage estimates from the MeanDRS dataset, Wade et al.[8] show that if SWOT meets its expected goal of observing rivers wider than 50-100m, the mission will likely capture 91.1%-95.8% of global river storage variability. This suggests that the scaled SWOT RSA we produce likely underestimates true storage variability in all global rivers by ~5-10% (i.e., by 20-40 km^3), notwithstanding the unobservability of water storage changes over frozen rivers in the winter.

3. Observational and methodological limitations

The hypsometric approach used in this study provides a simplified product as it leverages averaged width and WSE over 10-km long river reaches, removing the intra-reach spatial variability. The method also inherently assumes a symmetrical profile which only provides partial information on the true shape of the banks and extent of the floodplains. In addition, multi-channel structures are not resolved, and river deltas are not specifically processed to account for their complexity (e.g., anabranches) and the influence of ocean tides. We acknowledge that a refinement of the hypsometric approach would be needed for coastal areas. However, our approach provides the first sensible SWOT-based estimates of river storage changes with reasonable approximation at the global scale. More elaborate methodologies would require leveraging the SWOT geo-located pixel cloud product or fine-scale digital elevation models for better local accuracy, but would be impractical globally due to the very large data volume and the high computational resources involved.

SWOT's observational scope remains challenging to fully characterize, particularly in complex hydrological environments. The discrepancies observed between SWOT-derived and modeled RSA may originate, in part, from contrasted definitions of river storage between SWOT's observational perimeter and what models incorporate. While models may systematically integrate all water flowing through the river–floodplain continuum, SWOT's retrievals depend on surface water detection and altimetric retracking algorithms that may exclude or inconsistently capture key dynamic zones (particularly in the Version C RiverSP products). In areas like the Arctic wetlands and along snowy riverbanks in transitional periods, or in South America's Pantanal during the rainy season, SWOT's classification of river extent can vary significantly. More detailed and large-scale evaluation of SWOT river width detection will likely clarify how comparable these datasets truly are. By performing a tailored outlier analysis of the SWOT-derived RSA estimates, we also reveal occasional mismatches between the reaches' characteristics (topology, location of river centerline) and the ΔRSA estimates in complex riverine environments (e.g., braided systems and river deltas), highlighting limited confidence in these areas (see section 5 for more details).

There are several opportunities for the future improvement of our approach to derive river storage variability from SWOT observations. The relative recency of SWOT data product release presents a range

of challenges for accurate river storage estimation. At the time of this study, less than two full years of SWOT river observations are available, complicating the application of hypsometric methods. Furthermore, a significant portion of the available data is affected by quality issues, reducing the number of usable observations. We also find that river width measurements from SWOT are notably less reliable than those of water surface elevation. Although this discrepancy was anticipated[9], the increased uncertainty in width measurements affects the reliability of our hypsometric approach. In cases where the expected relationship between width and water surface elevation is not evident, we resort to fitting simplified rectangular hypsometric curves using median width values, which introduces additional uncertainty into the storage estimates. With future maturity of the SWOT data product and improvements in processing algorithms, the expected increase in data quality and availability will provide a stronger foundation for accurate river storage estimation from SWOT. Additionally, as the relationship between river width and WSE is frequently non-linear, we could fit more elaborate and appropriate piecewise linear or power-law relationships to more numerous SWOT observations of width and WSE.

Finally, the ~11-day average revisit frequency of SWOT – and even lower effective revisit frequency ~28 days after data quality filtering (Extended Data Fig. 1) – may also pose potential challenges to global storage estimation. Accurately capturing river variability, particularly during peak flow events, requires an observation frequency that significantly exceeds the temporal scale of typical flow events[10–12]. Previous studies have estimated that, as a result of its temporal sampling cadence, SWOT is expected to detect only about 55% of flood events[13]. When a flood event is not observed by SWOT at a given reach, the peak of the discharge hydrograph is not recorded, leading to an underestimation of the true magnitude of storage variability. This temporal sampling limitation may contribute to discrepancies observed between SWOT-derived storage anomalies and those simulated by global hydrological models[14], especially in short hydrographic networks like the Maritime continent.

4. Sensitivity of Active Riverbed Shapes and River Storage Anomaly (RSA) to SWOT Width and Elevation Uncertainties

Current recommendations (as of October 2025) from JPL and CNES are to avoid using widths uncertainties, while WSE uncertainties appear more reliable but are still under study. As it is still unknown whether the SWOT science requirements for width and elevation uncertainties have been met, we use in our main scenario an assumed WSE uncertainty of 0.1 m and a width uncertainty of 30 m applying the errors-in-variable approach, reflecting a favorable outcome for the SWOT mission measurement requirements. To document this methodological choice, we implemented various scenarios for input uncertainties in the FLaPE-Byrd software:

- Main scenario: WSE uncertainty = 0.1 m; Width uncertainty = 30 m.
- Alternate scenario 1: WSE uncertainty = 0.5 m; Width uncertainty = 30 m.
- Alternate scenario 2: WSE uncertainty = 0.1 m; Width uncertainty = 300 m.
- Alternate scenario 3: WSE uncertainty = 0.5 m; Width uncertainty = 300 m.

We ran the hypsometric and river storage anomaly workflows in the three alternate scenarios on ten contrasted basins, representative of different size, geomorphology, and hydroclimate, and for which initial results show consistent (Niger, Ob, Mississippi, Australia), good (Yangtze, Mekong), or seemingly degraded results (either in terms of seasonality or magnitude; Amazon, Lena, Nile, Western North and Central America).

We find that modifying the uncertainties used as input in the FLaPE-Byrd software does not influence the shapes of the active riverbeds and the resulting RSA time series, which remain inside a ±5% interval (Fig. S4). However, uncertainty of the inputs is propagated to uncertainty of the outputs. In the alternate scenario 1, with degraded WSE uncertainty of 0.5 m, the river storage variability uncertainty ΔRSA_u increases 3- to 4-fold compared to the main scenario, going from 173±16 km^3 to 168±55 km^3 for the Amazon basin. When increasing the width uncertainty to 300 m, the average river storage variability uncertainty ΔRSA_u increases 5- to 8-fold (alternate scenario 2), and 7- to 10-fold (alternate scenario 3, with concurrent degraded WSE uncertainty of 0.5 m), going from 173±16 km^3 to 174±84 km^3 and 173±119 km^3, respectively, for the Amazon basin. Propagated uncertainty disproportionately affects periods of high RSA, due to the

multiplicative interaction between river width and height (Fig. S4). These multiplicative factors are very stable across basins, indicating that the main RSA estimates are robust to input uncertainties, whose effects solely manifest through uncertainty propagation. As the mission progresses and uncertainties are better constrained, more consistent RSA uncertainties will be delivered.

5. Outlier Analysis of River Storage Variability (ΔRSA) from SWOT

The SWOT mission delivers the first satellite data products of their kind for surface water hydrology. The quality of the Level 2 KaRIn HR River Single Pass products depends directly on several innovative components, including prior river and lake databases, novel interferometric signal processing, and tailored extraction and filtering algorithms developed specifically for the mission. These frameworks and tools are improving incrementally as they are tested by JPL and CNES, and as the science community and mission users provide feedback. One of the major source of concern that is currently being addressed[15] is the topology and accuracy of the digital river network underlying the SWOT river vector products (i.e., the SWOT river database SWORD), such as the position of the river centerlines, which lie at the basis of the computation of width and elevation changes that we consider in this study.

Highly dynamic rivers, braided systems and coastal areas in particular are extremely complex to represent in a static river network database. Whether the SWOT river vector products accurately capture the correct range of surface water in these complex environments is still under study.

To analyze the consistency of our global ΔRSA estimates and the potential related issues with the SWORD database and data processing, we performed a global statistical analysis of the derived river storage variability estimates from SWOT with a tailored outlier detection method based on the expected increasing relationship between the range in cross-sectional area change $\Delta\delta A = \Delta RSA/L$ (we normalize ΔRSA by the varying length of each SWORD reach L to control for scaling) and the corresponding upstream drained area. For this, using all reaches in a given SWORD basin, we fit a linear regression of ΔδA on upstream drained area (obtained from the SWORD-MERIT translation[16]). We then flag as an outlier any reach for

which the residual of ΔδA compared to the regression-predicted value exceeds a heteroscedastic threshold (i.e., a non-uniform residual-based threshold), set as 2.5 standard deviations with a 0.1 growth rate of allowed residual (Fig. S5). This allows for a slightly larger variability in the ΔδA estimates to be tolerated with larger drained areas. Outlier flags therefore originate from suspicious SWOT data (high or low extreme values), unrealistic of the common values found in a similar range of upstream drained area in a specific basin. Suspicious reach data can be due to poor SWORD centerline quality, with too much or too little water pixels attributed to a reach, and/or unreliable signal processing, with degraded elevation retracking. However, we also acknowledge that outlier flags can be the result of strong tidal influence in river deltas, or erroneous values for upstream drained area in the SWORD-MERIT translation. Therefore, this outlier analysis overall highlights mismatches or limited coherence between the reaches' characteristics (topology, location of river centerline) and the ΔδA estimates derived from the associated SWOT measurements, i.e., the locations where our estimates may not be reliable.

3.4% of global river reaches are flagged as outliers, i.e., ~4200 out of ~125,000 reaches. We also find that 90% of the outliers are in the first three deciles of upstream drained area, and 60% in the first three deciles of distance from the outlet. This means that they are largely placed near coastal areas where channels can divide into a large number of anabranches, each contributing small flow accumulation. This is evidenced in Fig. S6-S8 and is consistent with the complexity in drawing SWORD centerlines and processing SWOT data in braided rivers, and especially in deltas like in the Ganges (Fig. S6). We also find that some inland braided/anabranching river systems show a significant number of outliers (e.g., the Niger inner delta, Fig. S7). Yet, some dynamic systems with fluctuating meanders like the Trinity river basin in Texas were not necessarily flagged as outliers (Fig. S8), showing a relative robustness of the water pixel assignment algorithm for the RiverSP products in challenging conditions. These less reliable estimates of RSA account for 8.8% of the global ΔRSA but impact average seasonality only by a 1.7-day shift.

Overall, we wish to underline the lower reliability of our RSA estimates in complex riverine environments (e.g., braided systems and river deltas), consistently with the lower robustness of SWORD and of current

SWOT data processing in these environments. Version D processing of SWOT data and future versions of SWORD (v17c, v18) will bring improvements to the reliability of SWOT river vector products in complex systems[15].

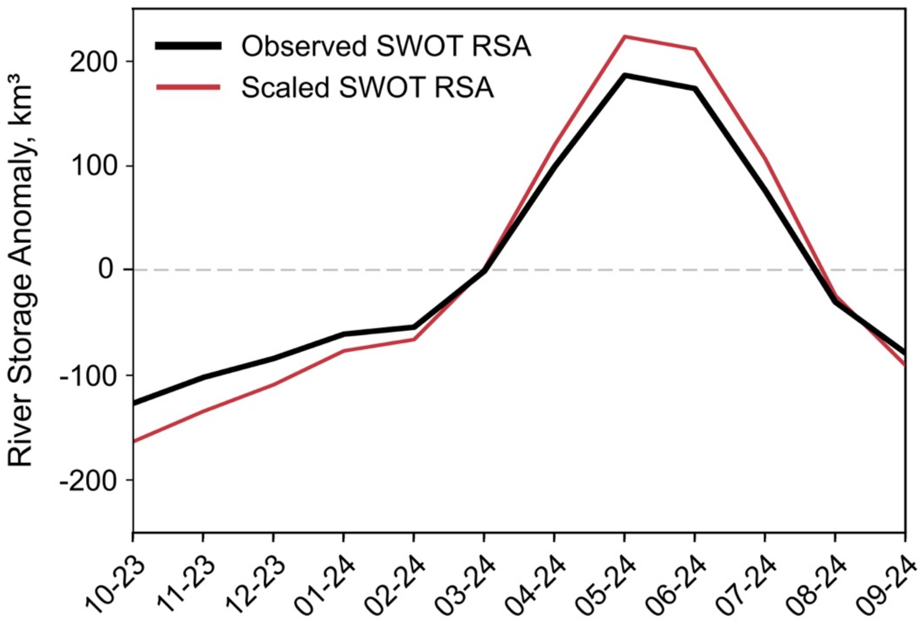

Fig. S1. Global SWOT-observed and SWOT-scaled river storage anomaly. SWOT-scaled river storage anomaly is adapted to account for SWORD reaches (Type 1 and 5) that are unobserved due to data filtering or lack of observations.

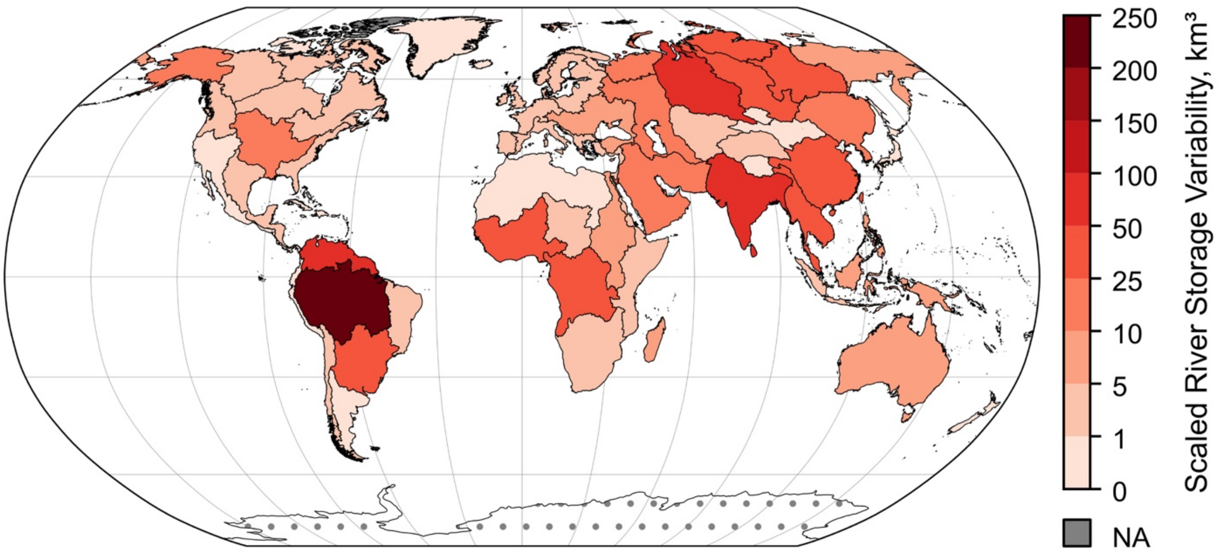

Fig. S2. Regional SWOT-scaled river storage variability, defined as the annual range of scaled monthly storage anomalies. Scaling of ΔRSA accounts for river storage anomaly unobserved by SWOT due to data filtering or lack of observations at SWORD reaches (Type 1 and 5), in each of the 61 Pfafstetter basins. Basemap from Natural Earth @ naturalearthdata.com.

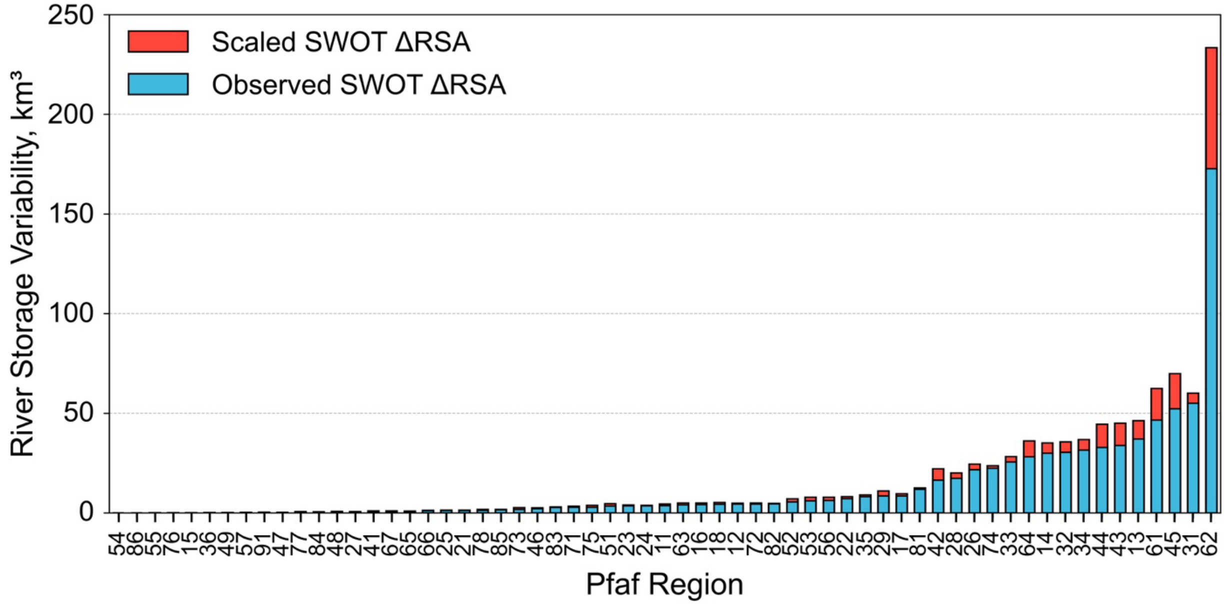

Fig. S3. Regional SWOT-observed and SWOT-scaled river storage variability. Scaling of ΔRSA accounts for river storage anomaly unobserved by SWOT due to data filtering or lack of observations at SWORD reaches (Type 1 and 5), in each of the 61 Pfafstetter basins.

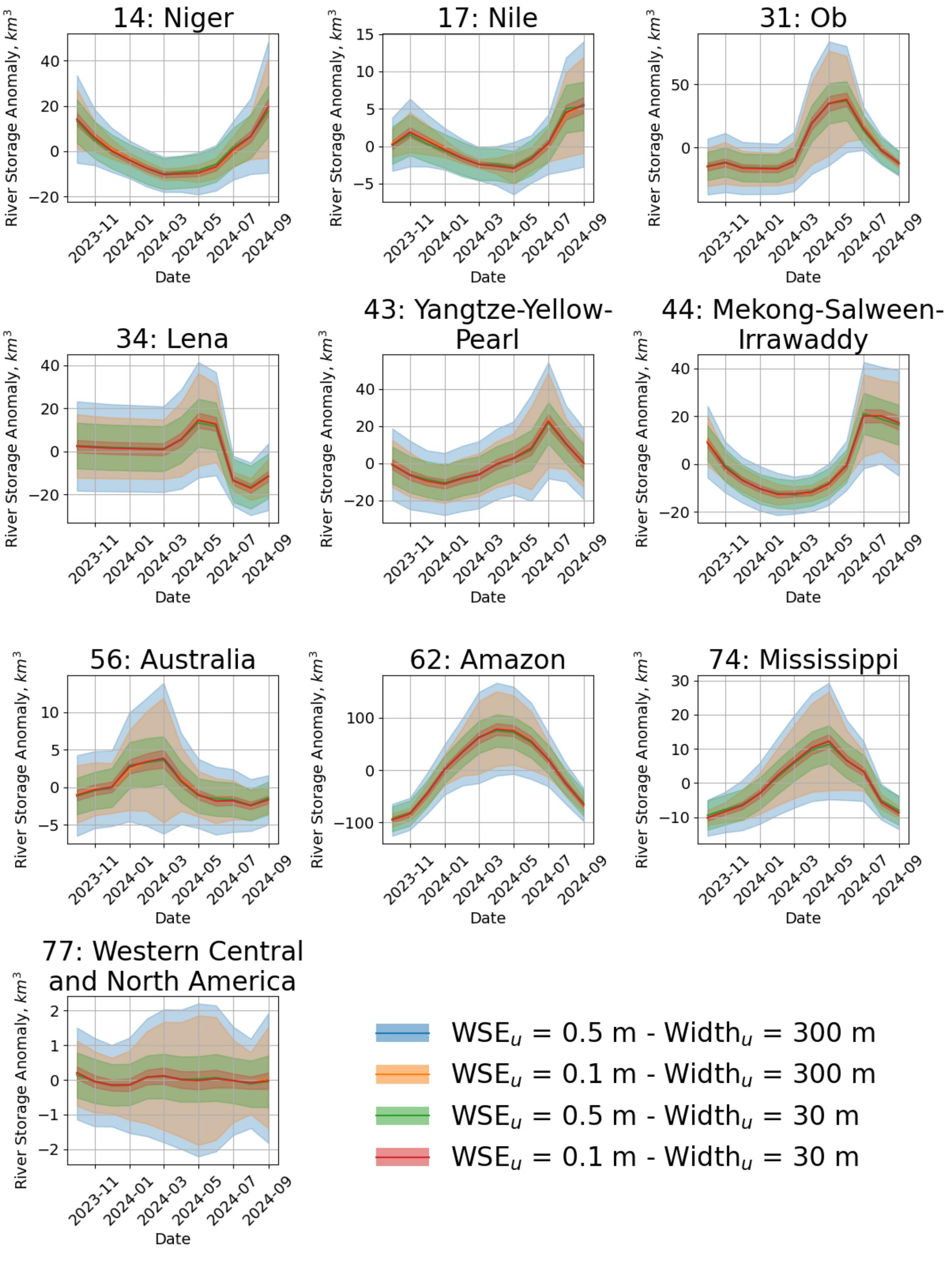

Fig. S4. SWOT river storage anomaly and its uncertainty under various input uncertainty scenarios. Ten contrasted basins are displayed under the main uncertainty scenario (red), and three additional degraded uncertainty scenarios (green, orange, and blue).

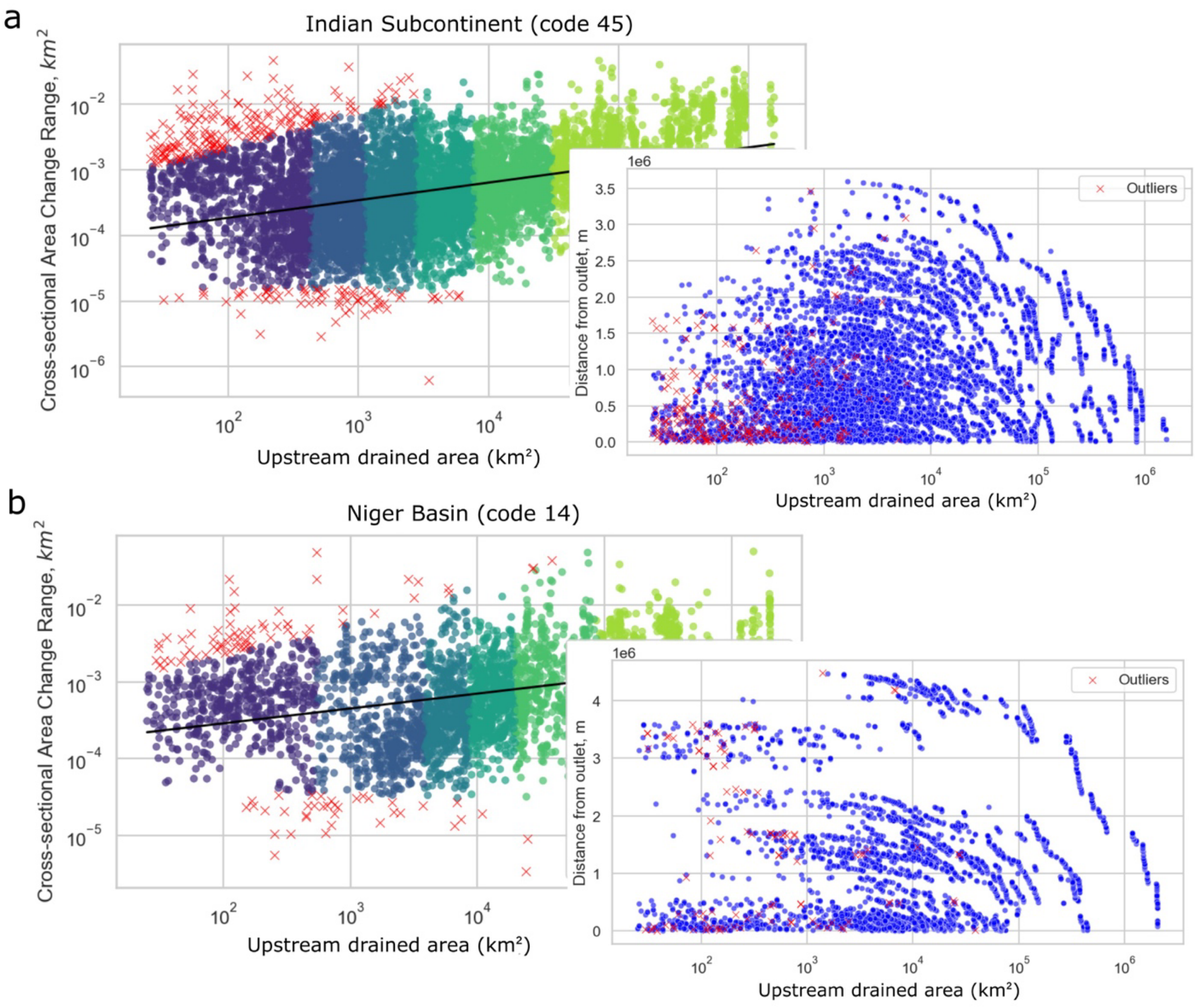

Fig. S5. Regression-based outlier detection identifying suspicious SWOT-observed cross-sectional area change range. Suspicious values of $\Delta\delta A = \Delta RSA/L$ are defined as extreme values within similar upstream drainage area per SWORD basin. Each dot/cross represents a SWORD river reach. Left plot is a scatterplot of $\Delta\delta A$ as a function of upstream drained area (both in log scale), with the associated regression line, and outlier identification (red crosses) based on a heteroscedastic threshold. Six upstream drained area bins are portrayed for illustration. An additional scatterplot in the bottom right shows the distance from the outlet as a function of upstream drained area for all reaches, showing outliers are mostly located in low drainage areas and close to river outlets. **a,** Indian Subcontinent (code 45). **b,** Niger River Basin (code 14).

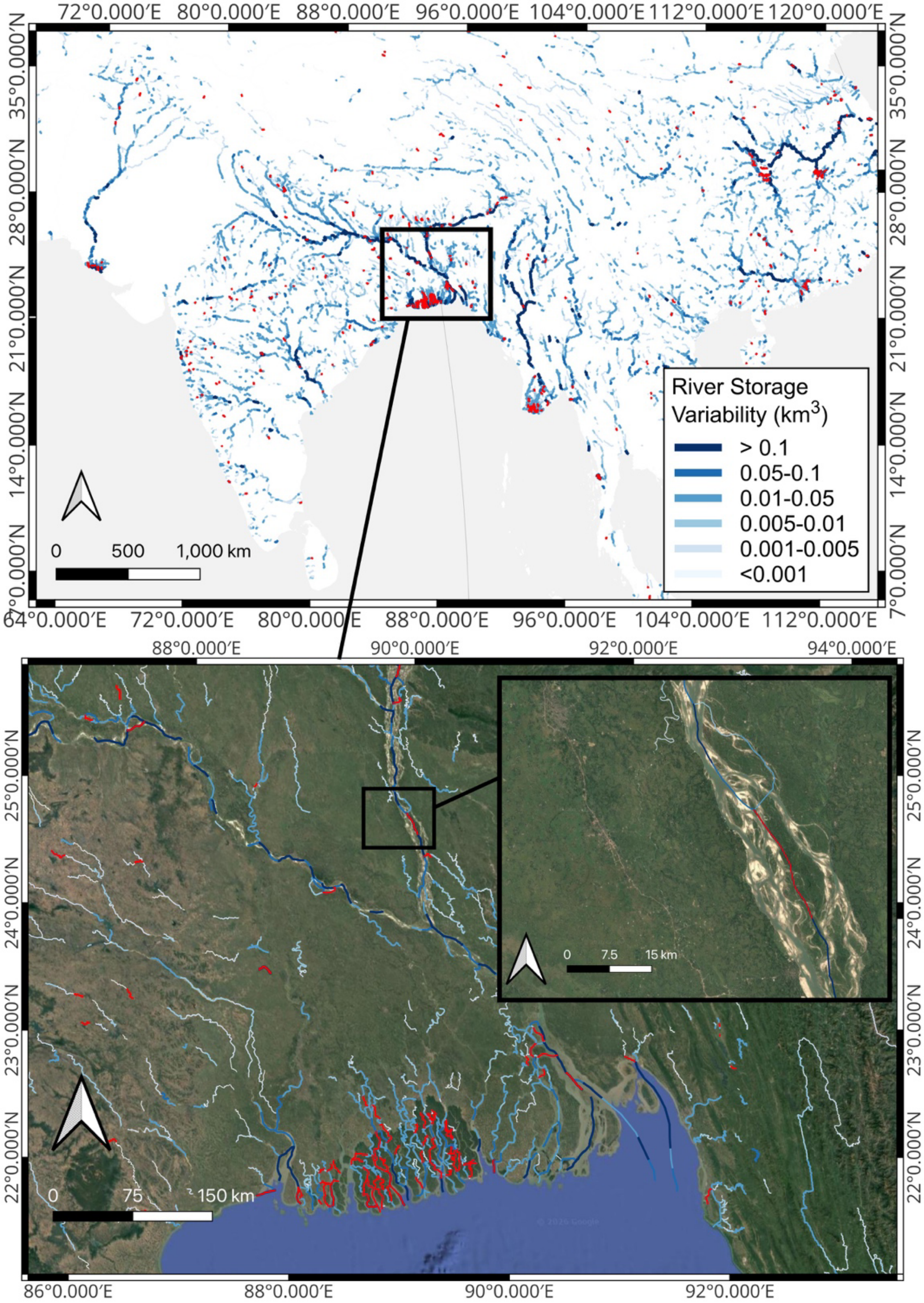

Fig. S6. Detection of SWOT-observed river storage variability outliers in South-Eastern Asia. Reaches in red indicate where the ΔδA estimate is considered an outlier with respect to the common values found in a similar range of upstream drained area, for a given SWORD basin. Basemap is courtesy of © 2026 Google Satellite Imagery, used for visualization purposes only (World Robinson Projection).

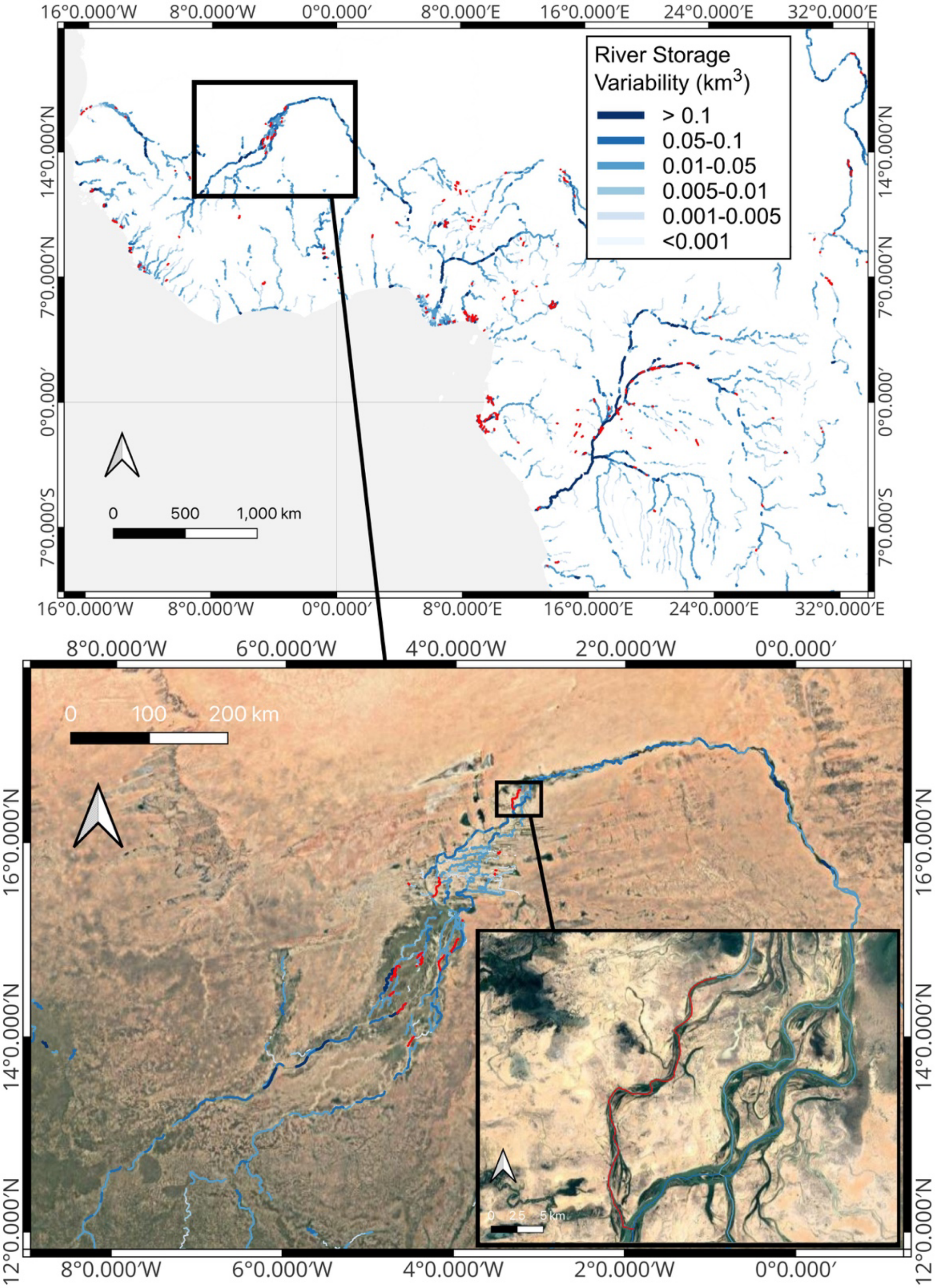

Fig. S7. Detection of SWOT-observed river storage variability outliers in Western Africa. Reaches in red indicate where the ΔδA estimate is considered an outlier with respect to the common values found in a similar range of upstream drained area, for a given SWORD basin. Basemap is courtesy of © 2026 Google Satellite Imagery, used for visualization purposes only (World Robinson Projection).

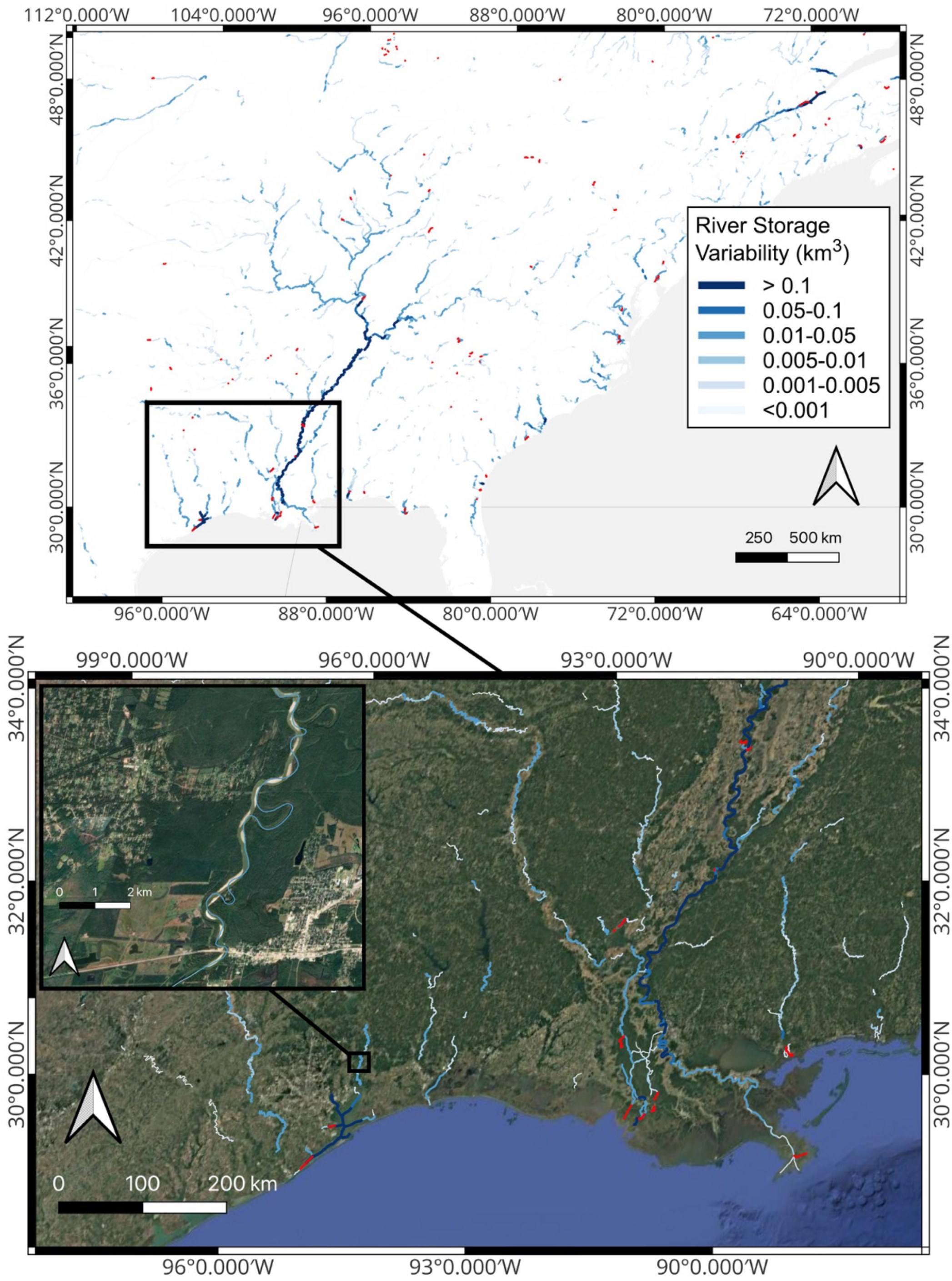

Fig. S8. Detection of SWOT-observed river storage variability outliers in Eastern North America. Reaches in red indicate where the ΔδA estimate is considered an outlier with respect to the common values found in a similar range of upstream drained area, for a given SWORD basin. Basemap is courtesy of © 2026 Google Satellite Imagery, used for visualization purposes only (World Robinson Projection).

References

1. Collins, E. L. *et al.* Global patterns in river water storage dependent on residence time. *Nat. Geosci.* 1–7 (2024) doi:10.1038/s41561-024-01421-5.
2. David, C. H. *et al.* River Network Routing on the NHDPlus Dataset. *Journal of Hydrometeorology* **12**, 913–934 (2011).
3. Yang, Y. *et al.* Global Reach-Level 3-Hourly River Flood Reanalysis (1980–2019). *Bulletin of the American Meteorological Society* **102**, E2086–E2105 (2021).
4. Yamazaki, D. *et al.* MERIT Hydro: A High-Resolution Global Hydrography Map Based on Latest Topography Dataset. *Water Resources Research* **55**, 5053–5073 (2019).
5. Lin, P. *et al.* Global Reconstruction of Naturalized River Flows at 2.94 Million Reaches. *Water Resources Research* **55**, 6499–6516 (2019).
6. MERIT Hydro Basins. reachhydro.org - MERIT-Basins. https://www.reachhydro.org/home/params/merit-basins (2019).
7. Altenau, E. H. *et al.* The Surface Water and Ocean Topography (SWOT) Mission River Database (SWORD): A Global River Network for Satellite Data Products. *Water Resources Research* **57**, e2021WR030054 (2021).
8. Wade, J. *et al.* Intrinsic Spatial Scales of River Stores and Fluxes and Their Relative Contributions to the Global Water Cycle. *Geophysical Research Letters* **52**, e2024GL113052 (2025).
9. Durand, M. *et al.* A Framework for Estimating Global River Discharge From the Surface Water and Ocean Topography Satellite Mission. *Water Resources Research* **59**, e2021WR031614 (2023).
10. Cerbelaud, A. *et al.* Peak Flow Event Durations in the Mississippi River Basin and Implications for Temporal Sampling of Rivers. *Geophysical Research Letters* **51**, e2024GL109220 (2024).
11. Cerbelaud, A. *et al.* Spatial Hydrographs of River Flow and Their Analysis for Peak Event Detection in the Context of Satellite Sampling. *Water Resources Research* **61**, e2024WR038444 (2025).

12. Cerbelaud, A. *et al.* Satellite Requirements to Capture Water Propagation in Earth's Rivers. *Reviews of Geophysics* **63**, e2024RG000871 (2025).
13. Frasson, R. P. de M., Schumann, G. J.-P., Kettner, A. J., Brakenridge, G. R. & Krajewski, W. F. Will the Surface Water and Ocean Topography (SWOT) Satellite Mission Observe Floods? *Geophysical Research Letters* **46**, 10435–10445 (2019).
14. Nickles, C. *et al.* How Does the Unique Space-Time Sampling of the SWOT Mission Influence River Discharge Series Characteristics? *Geophysical Research Letters* **46**, 8154–8161 (2019).
15. Collins, E. L. *et al.* River Network Routing and Discharge Partitioning on a Multichannel River Network. *Water Resources Research* **61**, e2025WR041417 (2025).
16. Wade, J. *et al.* Bidirectional Translations Between Observational and Topography-Based Hydrographic Data Sets: MERIT-Basins and the SWOT River Database (SWORD). *Water Resources Research* **61**, e2024WR038633 (2025).
